# Can the dual-rating regulation improve the rating quality of Chinese corporate bonds?

**DOI:** 10.1371/journal.pone.0259759

**Published:** 2021-12-02

**Authors:** Xiangyun Zhou

**Affiliations:** Shenzhen Institute of Information Technology, Shenzhen, China; University of Jyvaskyla, FINLAND

## Abstract

We developed a dual-reputational rating shopping model to introduce public and institutional reputations. Investor’s and regulator’s penalty rates are described as public and institutional reputations, respectively. We achieved the available conditions of single-rating and dual-rating regulations to prevent rating inflation in this model. To examine the regulatory effects of different types of regulations on Chinese corporate bond ratings, we utilize panel ordered logit models. Theoretical analysis and empirical tests show that, when the reputation effect is low, the single-rating regulation is better at improving rating quality, and when the reputation effect is high, the dual-rating regulation induces rating agencies to provide more accurate ratings. Compared to the regulatory effects of the single-rating and the multi-rating regulations, the dual-rating regulation most effectively improves the rating quality of corporate bonds and prevents rating inflation.

## Introduction

**Fig 1:**
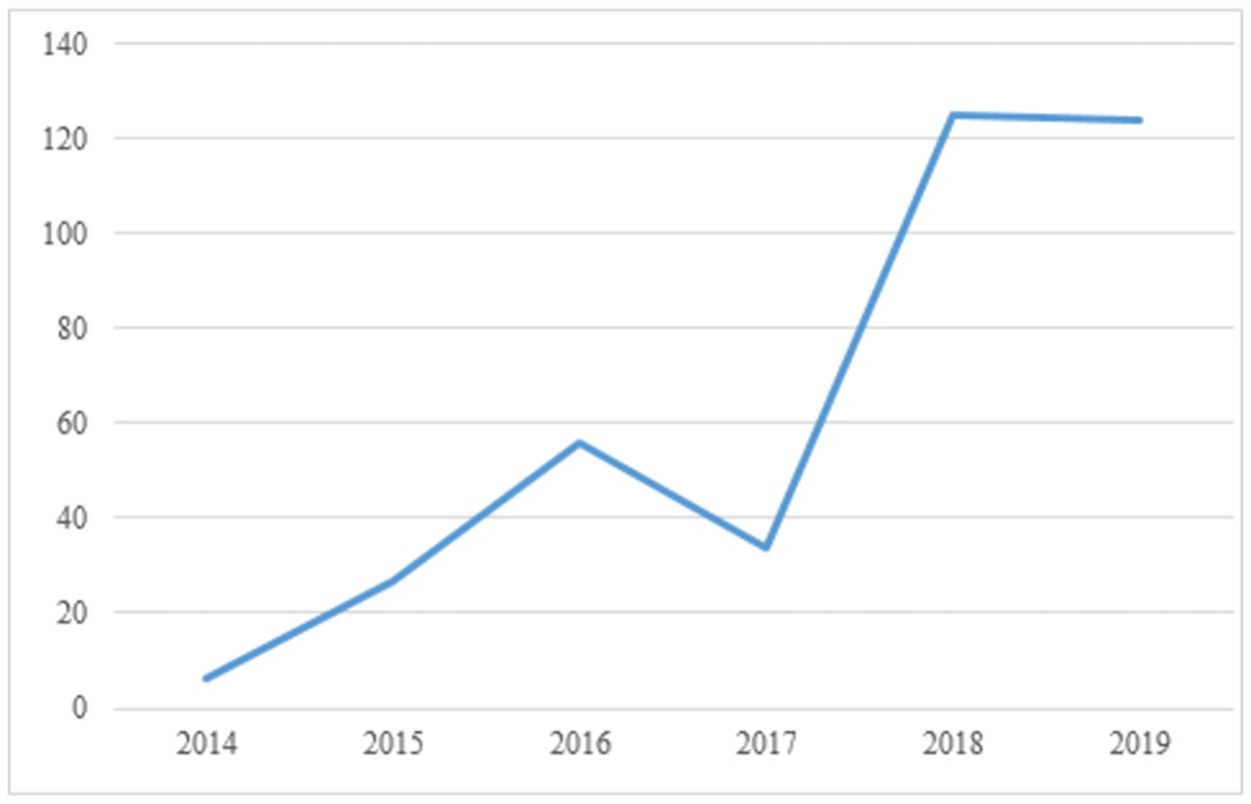
The number of defaults of Chinese corporate bonds from 2014 to 2019. Source: Wind Economic Database.

The continuous growth of the Chinese capital market, especially in the context of large-scale corporate bonds, necessitates a reliable rating system. The first default on Chinese corporate bonds occurred in 2014 when the “11 super-day debt” was not paid. Ever since—especially since 2018, when the Chinese corporate bonds market underwent some adjustments—defaults on corporate bonds have occurred frequently. Fig 1 shows the gradual increase in the number of defaults on Chinese corporate bonds from 2014 to 2018; the number decreased from 2016 to 2017, but then increased quickly from 2017 to 2018.

Statistics on the scale and proportion of different credit ratings of corporate bonds from 2011 to 2018 reveal that about 97.72% of corporate bonds were rated A- and above A- (Table 1). Based on the Wind Economic Database (https://www.wind.com.cn/en/edb.html), there are two hundred and three defaults on corporate bonds rated high ratings from 2014 to 2018 (Table 2). In December 2018 alone, there were one hundred and seventy-eight corporate bond defaults in total. Prior to these defaults, four corporate bonds were rated AAA; thirteen AA+; eighty-five AA and twenty-seven AA-. The high default rate on corporate bonds—72.47%—indicates inflated Chinese corporate bond ratings.

**Table 1:** The scale and the proportion of different credit ratings of corporate bonds.

Ratings	Scale	Proportion (%)
AAA+	7	0.06
AAA	3322	30.54
AAA-	137	1.26
AA+	2398	22.04
AA	3194	29.36
AA-	644	5.92
A+	417	3.83
A	345	3.17
A-	189	1.74
BBB+	69	0.63
BBB	14	0.13
BBB-	1	0.01
B and below B	142	1.31
Total	10879	100

Source: Wind Economic Database.

**Table 2:** The number of defaults on corporate bonds rated high ratings.

Year	The number of defaults
2018	129
2017	29
2016	28
2015	16
2014	1
Total	203

Source: Wind Economic Database.

The rating industry in the country had a late start, and earlier rating regulations were insufficient. However, Chinese rating regulators have now issued a series of regulations to promote the rating industry. On January 8, 2013, the People’s Bank of China (PBoC) issued self-discipline guidelines for debt financing instruments of non-financial enterprises. In the 6th provision of [1], the dual-rating regulation was encouraged for the rating market for the first time. On February 16, 2016, the National Association of Financial Market Institutional Investors (NAFMII) issued rating agencies’ market-evaluated rules for financing instruments of non-financial corporate debt. The 17th provision of [2] indicates that the NAFMII should punish—as in warn, publicly denounce—rating agencies providing inflated ratings. On December 26, 2019, the PBoC, the National Development and Reform Commission, the Ministry of Finance, and the China Securities Regulatory Commission jointly published the provisional rules of the administration of rating industry. The 26th provision of [3] proposes that in case of multiple ratings, rating agencies should follow these rules.

Regulators in the United States, England, and Japan have issued the dual-rating regulation to curb rating inflation. Similarly, since 2013, many regulatory institutions in China have introduced the dual-rating regulation to prevent the inflation of corporate bonds. Under this system, a corporate bond is rated by two rating agencies simultaneously; theoretically, this has certain reputational constraints in terms of cross-checking of ratings. However, the dual-rating regulation has not yet been officially implemented in China.

This study considers public and institutional reputations to improve the rating shopping model and discusses the conditions of the single-rating and dual-rating regulations. Additionally, we utilize the data on Chinese corporate bonds to conduct empirical tests. Our research attempts to answer two questions: Can the dual-rating regulation improve the rating quality of Chinese corporate bonds? How are the regulatory effects on different rating regulations?

The remainder of this article is structured as follows. Section 2 reviews the relevant literature and Section 3 constructs a dual-reputational rating shopping model. In Section 4, we theoretically analyze the conditions of single-rating and dual-rating regulations. In Section 5, we utilize panel ordered logit models to examine the regulatory effects on different rating regulations. Section 6 briefly discusses the implications of our findings and their prospects.

## Literature review

A few credit rating studies are based on the assumption of ratings shopping [4], that is, purchasing the best rating from a rating agency [5], which are inclined to provide inflated ratings for more revenue. Rating shopping, which may create conflicts of interest among rating agencies [6, 7], is an important hypothesis to combine with market structure [8].

As stated above, many countries have adopted the dual-rating regulation. Based on international experience, these regulations are important for order competition and healthy development in the rating industry. To the best of our knowledge, the United States first implemented the dual-rating regulation in 1936. According to [9, 10], the Office of the Comptroller of the Currency and the Federal Reserve issued the valuation principle according to which regulatory banks could not hold bonds that were not rated BBB or above by two credit rating agencies (CRAs). In July 2010, the Dodd-Frank Act allowed investors and corporations to estimate the accuracy of ratings and compare them with rating information issued by different CRAs. Japanese corporate bonds usually have two ratings: one can be provided by S&P or Moody’s, and the other should be issued by one of the two domestic CRAs [11]. After the 2008 subprime crisis, South Korea also adopted the dual-rating system.

A few researchers have discussed the effects of reputation on rating quality. Rating agencies have a dual-reputation effect, namely, public reputation and institutional reputation [12]. Regulators can impose penalties to induce rating agencies to improve their quality [13]. Meanwhile, if rating agencies provide inaccurate ratings, investors “vote with their feet” and dishonest CRAs lose their market share [14]. Thus, rating agencies are required to improve the rating quality both in terms of public reputation and institutional reputation [15]. Similarly, the entry of low-reputation CRAs may further inflate ratings [16]. When rating agencies have low reputations, bond issuers voluntarily announce more qualitative information to help investors evaluate bond risks [17].

The relationship between rating regulations and rating quality has been examined in a few studies. For example, the dual-rating regulation prevents the collusion of inflated ratings with the separation of economic cycles [10]. Regulatory initiatives to increase rating industry competition improve investment efficiency as long as corporate misreporting incentives are not significantly raised [18]. However, rating regulations may cause rating inflation [19]. Strict regulations force rating agencies to provide lower ratings [20] so that they can protect their reputations, which causes false warnings and unjustified rating downgrades [21].

Researchers differ on the issue of increased competition among rating agencies. Some opine that this induces rating agencies to provide more accurate information, which benefits investors [22]. Conversely, competition between small, local rating agencies and large, global rating agencies is not fair [23]. Interestingly, credit rating agencies are more likely to inflate ratings under duopoly than under monopoly [16]. Additionally, conflicts of interest may lead rating agencies to provide biased ratings [24].

While a few studies have focused on the rating shopping model to explore the effects of rating regulations on rating quality, others have studied the regulatory effects of different rating regulations. A few researchers have also shown that competition among rating agencies may lead to inflated ratings [16]. It is, therefore, important to discuss whether the dual-rating regulation can induce Chinese rating agencies to provide more accurate ratings.

The innovations of this study are as follows: First, unlike another study [25], we introduce the investor’s penalty rates and the regulator’s penalty rates to describe the dual-reputation and develop a dual-reputational rating shopping model. We use the improved model to analyze the available conditions of the single-rating and dual-rating regulations to prevent rating inflation. Second, we utilize panel ordered logit models to examine the effects of the single, dual, and multi-rating regulations on Chinese corporate bond ratings.

## The dual reputational rating shopping model

Based on the perspectives of expected revenue and regulatory cost, we developed a dual reputational rating shopping model to analyze the available conditions for the single-rating and dual-rating regulations.

**Fig 2:**
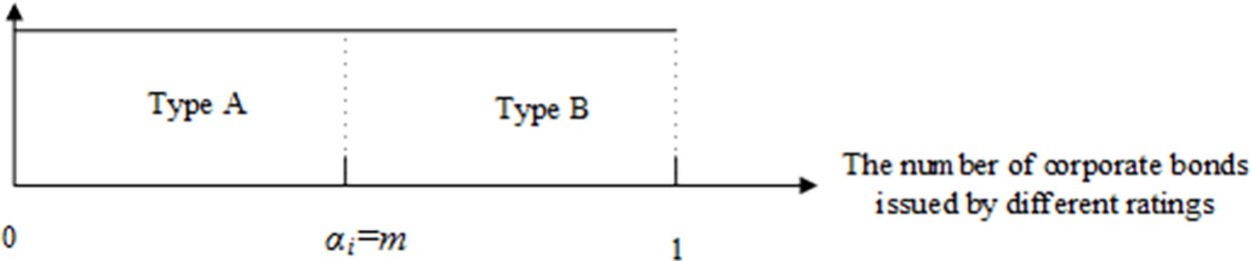
Rating accuracy.

**Fig 3:**
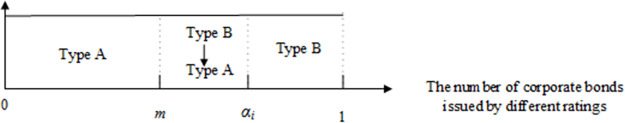
Rating inflation.

**Fig 4:**
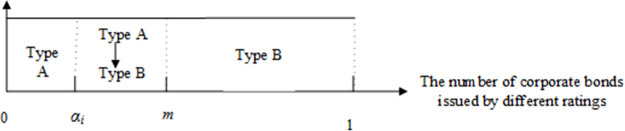
Rating deflation.

The assumptions of the model were similar to those of [10]. We suppose the multiplicity of rating agencies, issuers, investors, and regulators. All participants are risk-neutral. Issuers obtain financing from investors through a variety of corporate bonds [26]. Rating agencies can observe a corporate bond’s actual rating, but regulators and investors cannot. We suppose some variables of the regulator, rating agency *i*, and issuers are as follows:

1. There are two types of corporate bonds: A and B. The real proportion of type-A bonds is *m*, where *m* ∈ (0,1). In period 0, *z*_*i*_ denotes whether the regulator approves rating agency *i* to provide a rating service for issuers. If the regulator approves rating agency *i* in the rating industry, *z*_*i*_ = 1; otherwise, *z*_*i*_ = 0. *w*_*i*_ represents whether rating agency *i* can issues accurate ratings—If yes, *w*_*i*_ = 1; otherwise, *w*_*i*_ = 0. The regulator has approval *c*_*A*_ and regulatory costs *c*_*M*_ for each rating agency.
2. The rating fee for the rating agency *i* is *f*_*i*_. The rating threshold of type-A corporate bonds is *α*_*i*_, namely, the number of type-A corporate bonds issued by rating agency *i*. As a result of rating shopping, there is a slight difference between rating threshold *α*_*i*_ and the real proportion of type-A bonds *m*. As shown in Figs 2–4, we describe the rating accuracy, rating inflation and rating deflation.
3. An issuer’s utility of an A rating is *φ*, where *φ* > 0, whereas the issuer’s utility of a B rating is 0. *D*_*i*_ is the demand function of rating agency *i*. When rating agency *i* is monopolistic, $D_{i}^{}=\left\{\begin{array}{c} \alpha_{i}^{},f_{i}^{}<\varphi \\ 0,f_{i}^{}>\varphi \end{array}\right.$. We suppose that other rating agencies have the same rating fees *f*_−*i*_ and an identical rating threshold *α*_−*i*_. In the competition of *n* rating agencies, the demand function of rating agency *i* is:
$$D_{i}^{}=\left\{\begin{array}{c} \alpha_{i}^{},f_{i}^{}<f_{-i}^{},f_{i}^{}<\varphi \\ \frac{1}{n}\alpha_{i}^{},f_{i}^{}=f_{-i}^{}\leq \varphi ,\alpha_{i}^{}<\alpha_{-i}^{}\\ \alpha_{i}^{}-\frac{n-1}{n}\alpha_{-i}^{},f_{i}^{}=f_{-i}^{}\leq \varphi ,\alpha_{i}^{}>\alpha_{-i}^{}\\ 0,otherwise \end{array}\right.$$ (1)

From Eqs (2) and (3), the expected revenue function of rating agency *i* and the cost function of the regulator are constructed by [25], where *σ*_*RA*_ and *σ*_*RE*_ represent the discount factors of rating agency *i* and the regulator, respectively.


$$R_{RA}=(1-\sigma_{RA})\sum_{t=1}^{\infty }\sigma_{RA}^{t-1}D_{i}f_{i}$$ (2)

$$c_{RE}=(1-\sigma_{RE})\sum_{t=1}^{\infty }\sigma_{RE}^{t-1}[c_{A}\sum_{i=1}^{n}z_{i}+c_{M}\sum_{i=1}^{n}w_{i}]$$ (3)

In this model, we ignore the dual reputation of rating agencies in the expected revenue function of rating agency i and the regulator’s cost function [25]. Meanwhile, it is necessary to discuss the effect of rating regulations on ratings and analyze the conditions of different rating regulations to prevent rating inflation. According to [10, 20], we replace discount factors with the investor’s penalty rates and regulator’s penalty rates. Rating agencies can be motivated to improve rating quality through the reputation effect. The dual reputational rating shopping model is:

In this model, we ignore the dual reputation of rating agencies in the expected revenue function of rating agency *i* and the regulator’s cost function [25]. Meanwhile, it is necessary to discuss the effect of rating regulations on ratings and analyze the conditions of different rating regulations to prevent rating inflation. According to [10, 20], we replace the discount factors *σ* with the investor’s penalty rates *ρ*_*RA*_ and regulator’s penalty rates *ρ*_*RE*_. Rating agencies can be motivated to improve rating quality through the reputation effect. The dual reputational rating shopping model is:

$$R_{RA}=(1-\rho_{RA})\cdot \sum_{t=1}^{\infty }\rho_{RA}^{t-1}D_{i}f_{i}$$ (4)

$$c_{RE}=(1-\rho_{RE})\sum_{t=1}^{\infty }\rho_{RE}^{t-1}(c_{A}\sum_{i=1}^{n}z_{i}+c_{M}\sum_{i=1}^{n}w_{i})$$ (5)

## Theoretical analysis of two rating regulations

Based on the 6th provision of [1], we suppose that the regulator designs two regulatory strategies, namely, the single-rating regulation and the dual-rating regulation. We analyze the conditions of the two rating regulations from the perspectives of expected revenue and regulatory costs.

### The single-rating regulation

In period 0, the regulator approves only one rating agency to provide ratings. The rating fee of rating agency is *ϕ*_*i*_ and the rating threshold is *α*_*i*_. Subsequently, if the rating accuracy of rating agency *i* is lower than that of rating agency *j*, it will be replaced by rating agency *j* in next period.

According to Eq (4), when *α*_*i*_ = *m*, rating agencies provide accurate ratings. The expected revenue of the rating agency *i* is:

$$R_{1}=(1-\rho_{RA})(\sum_{t=1}^{\infty }\rho_{RA}^{t-1}m\phi_{i})=m\phi_{i}$$ (6)

When *α*_*i*_ > *m*, rating agencies provide inflated ratings. Owing to the single-rating regulation, the regulator approves only one rating agency to issue ratings. When *t* = 1 and *α*_*i*_ = 1, the expected revenue of the rating agency *i* is the highest. In this situation, the expected revenue of the rating agency *i* is:

$$R_{2}=(1-\rho_{RA})(1\cdot \phi_{i}+\sum_{t=2}^{\infty }\rho_{RA}^{t-1}\cdot 0)=(1-\rho_{RA})\cdot \phi_{i}$$ (7)

When *α*_*i*_ < *m*, rating agencies issue deflated ratings. In this situation, many issuers may decrease rating demands. Only when the rating threshold *α*_*i*_ is the real proportion of type-A bonds can the issuers pay for the rating service. In this situation, the expected revenue of the rating agency *i* is:

$$R_{3}=(1-\rho_{RA})(\sum_{t=1}^{\infty }\rho_{RA}^{t-1}\alpha_{i}\phi_{i})=\alpha_{i}\phi_{i}$$ (8)

According to Eq (5), the regulatory cost of the single-rating regulation is:

$$c_{1}=(1-\rho_{RE})[c_{A}+\sum_{t=2}^{\infty }\rho_{RE}^{t-1}c_{A}+\sum_{t=1}^{\infty }\rho_{RE}^{t-1}c_{M}]=c_{A}+c_{M}$$ (9)

### The dual-rating regulation

In period 0, the regulator requires issuers to buy two ratings from the two rating agencies. We suppose that rating agencies *i* and *j* have different reputational rankings in rating industry, and their rating thresholds are also different. The rating threshold of rating agency *j* is *m*. The rating fees for rating agency *i* and rating agency *j* are *ϕ*_*i*_. If the rating accuracy of rating agency *i* is low, other rating agencies will replace it. In this situation, the demand function of rating agency *i* is:

$$D_{i}=\left\{\begin{array}{c} \alpha_{i},f_{i}<\phi_{i}\\ \frac{1}{2}\alpha_{i},f_{i}=f_{j}=\phi_{i},\alpha_{i}\leq \alpha_{j}\\ \alpha_{i}-\frac{1}{2}\alpha_{j},f_{i}=f_{j}=\phi_{i},\alpha_{i}>\alpha_{j}\\ 0,otherwise \end{array}\right.$$ (10)

The reputational ranking of rating agency *i* is higher than that of rating agency *j*. In this situation, the rating agency *i* issues accurate ratings, namely, *α*_*i*_ = *m*, whereas rating agency *j* provides inflated ratings, namely, *α*_*j*_ > *m*. The rating fees of the two rating agencies are *ϕ*_*i*_. According to Eq (10), the demand function of rating agency *i* is $D_{i}=\frac{m}{2}$, and the expected revenue of rating agency *i* is:

$$R_{4}=(1-\rho_{RA})\sum_{t=1}^{\infty }\rho_{RA}^{t-1}\frac{m}{2}\phi_{i}=\frac{m}{2}\phi_{i}$$ (11)

The reputational ranking of rating agency *i* is lower than that of rating agency *j*. In this situation, the rating agency *i* issues inflated ratings, namely, *α*_*i*_ > *m*, whereas rating agency *j* provides accurate ratings, namely, *α*_*j*_ = *m*. The rating fees of the two agencies are *ϕ*_*i*_. According to Eq (10), the demand function of rating agency *i* is $D_{i}=\alpha_{i}-\frac{m}{2}$, and the expected revenue of rating agency *i* is:

$$R_{5}=(1-\rho_{RA})[(\alpha_{i}-\frac{m}{2})\cdot \phi_{i}+\sum_{t-2}^{\infty }\rho_{RA}^{t-1}\cdot 0]=(1-\rho_{RA})\cdot (\alpha_{i}-\frac{m}{2})\cdot \phi_{i}$$ (12)

According to Eq (5), the regulatory cost of the dual-rating regulation is:

$$c_{2}=(1-\rho_{RE})[2c_{A}+\sum_{t-1}^{\infty }\rho_{RE}^{t-1}2c_{M}]=2c_{A}\cdot (1-\rho_{RE})+2c_{M}$$ (13)

#### Proposition 1

There exists an investors’ penalty rate *ρ*_*RA*_ ∈(1−*m*, 1] and a regulator’s penalty rate $\rho_{RE}\in (0,\frac{c_{A}+c_{M}}{2c_{A}})$. In this situation, the single-rating regulation is effective in improving rating quality. However, because of low reputation effect, the dual-rating regulation is ineffective in checking rating inflation.

Proof 1 can be seen in S1 Appendix.

#### Proposition 2

There exists an investors’ penalty rate $\rho_{RA}\in (\frac{2(\alpha_{i}-m)}{2\alpha_{i}-m},1]$ and a regulator’s penalty rate $\rho_{RE}\in (\frac{c_{A}+c_{M}}{2c_{A}},1)$. In this situation, the regulatory effect of the dual-rating regulation is better than that of the single-rating regulation.

Proof 2 can be seen in S1 Appendix.

## Empirical analysis

As shown in the propositions of the dual reputational rating shopping model, dual reputation has an effect on different rating regulations. When dual reputation is in available conditions, there are a few differences between the regulatory effects of the single-rating and dual-rating regulations.

The credit rating variable is an ordinal and qualitative variable. The traditional linear transformation of ratings will not hold here; because of the boundaries of rating symbols, this type of transformation will cause errors when large samples are employed. An ordered logit model can avoid this problem. Based on [21, 27], a few researchers have utilized the ordered logit model to study corporate bond ratings. The construction of the panel ordered logit model is shown in [28]. Therefore, we utilize panel ordered logit models to examine the regulatory effects of the single, dual, and multi-rating regulations on Chinese corporate bond ratings.

### Description of the data and sample

We collected data on 10,876 Chinese corporate bond ratings from 2011 to 2018. There are 3,730 corporate bonds rated by only one agency; 4,108 by two agencies; and 3,038 by three agencies. During this period, 908 corporate bonds were rating upgrades; 3,038 were downgrades. In Table 3, all the variables are described.

**Table 3:** The description of variables.

The efforts of rating agencies	The number of rating outlook (positive, negative, unchanged) and rating changes (rating upgrades and rating downgrades).
The rating defaults	It is a dummy variable. If rating default occurs, it will be 1; otherwise, 0.
The rating scores of corporate bonds	Chinese corporate bond ratings are ordinal variables and are given values ranging from 1 (for AAA+) to 23 (for D).
The issuing year of corporate bonds	The log of issuing year of corporate bonds
The coupon rate	The issuing coupon rate
The types of rating agencies	It is a dummy variable. If the rating agency is domestic rating agencies, it will be 1; otherwise, 0.
Industry dummy	The dual-rating regulation is for non-financial corporate bonds. If issuers are non-financial, it will be 1; otherwise, 0.

We utilized the total number of rating outlooks and rating changes to describe the efforts of rating agencies. Where no rating outlook or rating changes was provided by the rating agencies, the variable was 0; otherwise, 1. If both rating outlook and rating change was provided, it was 2.

Reputation-wise, domestic rating agencies (Dagong, Chengxin, Jincheng, Lianhe, and Pengyuan) and global-partnered ones (Brilliance, Chengxin–Moody, Lianhe–Fithch) have many differences [27]. As a result of technical partnerships, the reputation effect of the latter is higher.

As shown in Table 4, the mean and maximum efforts of rating agencies were 0.203 and 2, respectively, which indicates significant differences among rating agencies. The mean rating default is 0.010, which demonstrates that a few rating defaults can occur. The average rating scores of corporate bonds provided by one, two, and three rating agencies were 4.665, 3.896, and 3.602, respectively. We identified that more the number of rating agencies providing ratings for a corporate bond, lower the mean rating score and higher the rating. The standard error of the rating scores provided by two rating agencies is the smallest, which shows that the rating quality of the dual-rating regulation is the best. The mean issuing year of corporate bonds is 0.651, and the mean of the coupon rate is 0.056. The characteristics of rating agencies and corporate bonds are the types of rating agencies and industry dummy. The mean of the type of rating agencies is 0.787, and the mean of the industry dummy is 0.899. This shows that the number of domestic rating agencies is greater than that of global-partnered rating agencies. Most non-financial corporate bonds exist in China.

**Table 4:** The statistics of variables.

Variables	Maximum	Minimum	Mean	Standard error
The efforts of rating agencies	2	0	0.203	0.456
The rating default	1	0	0.001	0.030
The rating scores of corporate bonds provided by one rating agency	21	2	4.665	2.491
The rating scores of corporate bonds provided by two rating agencies	21	2	3.896	2.295
The rating scores of corporate bonds provided by three rating agencies	21	2	3.602	2.505
The issuing year of corporate bonds	1.180	0	0.651	0.139
The coupon rate	0.100	0	0.056	0.014
The types of rating agencies	1	0	0.787	0.410
Industry dummy	1	0	0.899	0.301

Source: Wind Economic Database.

### Empirical results

We utilize panel ordered logit models to analyze the effects of different rating regulations on rating upgrades and downgrades. The dependent variables include rating upgrades and rating downgrades, which are dummy variables. If rating agencies provide rating upgrades or rating downgrades, it will be equal to 1; otherwise, 0. Due to the lack of direct variables to describe different rating regulations, we chose the rating scores of corporate bonds provided by one rating agency, and the average rating scores provided by two and three rating agencies, as proxy variables.

Table 5 reports the effects of the single, dual, and multi-rating regulations on rating upgrades. In the Column 1 model, the coefficient of the rating scores provided by one rating agency is significantly negative in S1 Data. In the Column 2 model, the coefficient of the average rating scores provided by two rating agencies is also significantly negative in S2 Data. However, in the Column 3 model, the coefficient of the average rating scores provided by three rating agencies is significantly positive in S3 Data. The results show that agencies provide fewer rating upgrades under the single-rating and dual-rating regulations compared to the multi-rating regulation. Additionally, the coefficient of the rating scores provided by one rating agency is bigger than that of the average rating scores provided by two rating agencies, which indicates that the latter can prevent rating inflation.

**Table 5:** Regression results of rating upgrades.

Variables	Ordered Logit (1)	Ordered Logit (2)	Ordered Logit (3)
The rating scores of corporate bonds provided by one rating agency	-0.226*** (0.043)		
The average rating scores of corporate bonds provided by two rating agencies		-0.048* (0.032)	
The average rating scores of corporate bonds provided by three rating agencies			0.040* (0.022)
The issuing year of corporate bonds	-1.035*** (0.509)	0.254 (0.410)	3.328*** (0.449)
The coupon rate	-1.263 (4.837)	4.036 (4.768)	25.938*** (5.127)
The types of rating agencies	0.258 (0.166)	-0.143 (0.136)	-0.067 (0.147)
Industry dummy	Yes	Yes	Yes
*C*_*1*_	2.015 (0.478)	3.421(0.424)	6.876 (0.491)

Table 5 reports that the numbers in the parentheses are standard errors.

*, **, *** denote that the coefficient is statistically significant at the 10%, 5%, 1% levels respectively.

Table 6 reports the effects of the single, dual, and multi-rating regulation on rating downgrades. In the Column 1 model, the coefficient of the rating scores provided by one rating agency is significantly positive in S1 Data. In the Column 2 model, the coefficient of the average rating scores provided by two rating agencies is significantly positive in S2 Data. In the Column 3 model, the coefficient of the average rating scores provided by three rating agencies is also significantly positive in S3 Data. Additionally, the coefficient of the average rating scored provided by two rating agencies is the smallest. The results suggest that the three rating regulations are effective in decreasing rating deflation. Based on Tables 5 and 6, the regulatory effects of the dual-rating regulation is the best and rating agencies can improve rating quality under this regulation.

**Table 6:** Regression results of rating downgrades.

Variables	Ordered Logit (1)	Ordered Logit (2)	Ordered Logit (3)
The rating scores of corporate bonds provided by one rating agency	0.518*** (0.047)		
The average rating scores of corporate bonds provided by two rating agencies		0.458*** (0.039)	
The average rating scores of corporate bonds provided by three rating agencies			0.557*** (0.053)
The issuing year of corporate bonds	5.629*** (1.414)	4.104*** (1.048)	5.943*** (1.504)
The coupon rate	87.950*** (16.273)	47.911*** (12.515)	70.782*** (17.797)
The types of rating agencies	15.474 (822.423)	2.502*** (0.751)	0.281 (0.571)
Industry dummy	Yes	Yes	Yes
*C*_*1*_	33.204 (822.425)	15.252 (1.477)	16.725 (1.905)

Table 6 reports that the numbers in the parentheses are standard errors.

*, **, *** denote that the coefficient is statistically significant at the 10%, 5%, 1% levels respectively.

In Table 7, we discuss whether different rating regulations can improve the efforts of rating agencies. We select the effort of rating agencies as an indicator of regulatory effectiveness, which is the dependent variable. In the Column 1 model, the coefficient of the rating scores provided by one rating agency is significantly positive in the S1 Data. The effort of rating agencies was 0.542 ($\frac{e^{0.167}}{1+e^{0.167}}$) under the single-rating regulation. In the Column 2 model, the coefficient of the average rating scores provided by two rating agencies is significantly positive in the S2 Data. The effort of the rating agencies is 0.556 ($\frac{e^{0.225}}{1+e^{0.225}}$) under the dual-rating regulation. In the Column 3 model, the coefficient of the average rating scores provided by three rating agencies is also significantly positive in the S3 Data. The effort of rating agencies is 0.548 ($\frac{e^{0.192}}{1+e^{0.192}}$) under the multi-rating regulation.

**Table 7:** Regression results of effort of rating agencies.

Variables	Ordered Logit (1)	Ordered Logit (2)	Ordered Logit (3)
The rating score of corporate bonds issued by one rating agency	0.167*** (0.016)		
The average rating score of corporate bonds issued by two rating agencies		0.225*** (0.018)	
The average rating score of corporate bonds issued by three rating agencies			0.192*** (0.017)
The issuing year of corporate bonds	-0.424 (0.393)	0.525* (0.315)	1.732*** (0.323)
The coupon rate	-14.401*** (3.459)	6.646*(3.418)	26.580*** (3.670)
The type of rating agencies	0.369*** (0.133)	0.247** (0.109)	0.221** (0.111)
Industry dummy	Yes	Yes	Yes
*C*_*1*_	2.731 (0.364)	4.316 (0.332)	5.632 (0.351)
*C*_*2*_	5.121 (0.387)	6.506 (0.348)	8.321 (0.379)

Table 7 reports that the numbers in the parentheses are standard errors.

*, **, *** denote that the coefficient is statistically significant at the 10%, 5%, 1% levels respectively.

The results demonstrate that the effort of rating agencies is the highest for the dual-rating regulation, and rating agencies can improve their efforts to provide more rating information under different rating regulations.

In Table 8, we investigate whether different rating regulations can decrease rating defaults. We consider rating default to be another indicator of regulatory effectiveness. In the Column 1 model, the coefficient of the rating scored provided by one rating agency is significantly positive in the S1 Data. The probability of rating defaults is 0.612 ($\frac{e^{0.455}}{1+e^{0.455}}$) under the single-rating regulation. In the Column 2 model, the coefficient of the average rating scores provided by two rating agencies is significantly positive in the S2 Data. The probability of rating defaults is 0.596 ($\frac{e^{0.389}}{1+e^{0.389}}$) under the dual-rating regulation. In the Column 3 model, the coefficient of the average rating scores provided by three rating agencies is also significantly positive in the S3 Data. The probability of rating defaults is 0.620 ($\frac{e^{0.491}}{1+e^{0.491}}$) under the multi-rating regulation.

**Table 8:** Regression results of rating defaults.

Variables	Ordered Logit (1)	Ordered Logit (2)	Ordered Logit (3)
The rating scores of corporate bonds provided by one rating agency	0.455*** (0.054)		
The average rating scores of corporate bonds provided by two rating agencies		0.389*** (0.032)	
The average rating scores of corporate bonds provided by three rating agencies			0.491*** (0.088)
The issuing year of corporate bonds	-1.706 (2.544)	3.708** (1.685)	1.354 (1.745)
The coupon rate	81.289** (39.757)	29.057 (20.275)	39.696 (26.326)
The types of rating agencies	13.874 (1844.995)	0.375 (0.617)	0.232 (0.943)
Industry dummy	Yes	Yes	Yes
*C*_*1*_	-29.1618 (1844.997)	-12.9243 (2.062)	-15.1496 (2.911)

Table 8 reports that the numbers in the parentheses are standard errors.

*, **, *** denote that the coefficient is statistically significant at the 10%, 5%, 1% levels respectively.

The results show that the probability of rating defaults is the lowest under the dual-rating regulation, and rating agencies can decrease rating defaults under different rating regulations.

### Robust test

To further illustrate the robustness of the results, we utilized the regression results for the models that did not include control variables as the control group [28]. We directly examined the effects of different rating regulations on rating upgrades, rating downgrades, the efforts of rating agencies, and rating defaults.

As shown in Tables 5 to 12, the coefficients of the rating scores of corporate bonds provided by one rating agency, the average rating scores of corporate bonds provided by two rating agencies, and the average rating scores of corporate bonds provided by three rating agencies are different, but the signs and significance levels are the same. These findings indicate that the regression results of rating upgrades, rating downgrades, the efforts of rating agencies, and rating defaults are robust.

**Table 9:** Robust results of rating upgrades.

Variables	Ordered Logit (1)	Ordered Logit (2)	Ordered Logit (3)
The rating scores of corporate bonds provided by one rating agency	-0.907*** (0.114)		
The average rating scores of corporate bonds provided by two rating agencies		-0.084* (0.098)	
The average rating scores of corporate bonds provided by three rating agencies			0.2000** (0.023)

Table 9 reports that the numbers in the parentheses are standard errors.

*, **, *** denote that the coefficient is statistically significant at the 10%, 5%, 1% levels respectively.

**Table 10:** Robust results of rating downgrades.

Variables	Ordered Logit (1)	Ordered Logit (2)	Ordered Logit (3)
The rating scores of corporate bonds provided by one rating agency	4.693*** (0.245)		
The average rating scores of corporate bonds provided by two rating agencies		4.242*** (0.242)	
The average rating scores of corporate bonds provided by three rating agencies			7.049** (0.394)

Table 10 reports that the numbers in the parentheses are standard errors.

*, **, *** denote that the coefficient is statistically significant at the 10%, 5%, 1% levels respectively.

**Table 11:** Robust results of effort of rating agencies.

Variables	Ordered Logit (1)	Ordered Logit (2)	Ordered Logit (3)
The rating scores of corporate bonds provided by one rating agency	0.033*** (0.019)		
The average rating scores of corporate bonds provided by two rating agencies		0.173*** (0.019)	
The average rating scores of corporate bonds provided by three rating agencies			0.047** (0.023)

Table 11 reports that the numbers in the parentheses are standard errors.

*, **, *** denote that the coefficient is statistically significant at the 10%, 5%, 1% levels respectively.

**Table 12:** Robust results of rating defaults.

Variables	Ordered Logit (1)	Ordered Logit (2)	Ordered Logit (3)
The rating scores of corporate bonds provided by one rating agency	0.497*** (0.052)		
The average rating scores of corporate bonds provided by two rating agencies		0.395*** (0.027)	
The average rating scores of corporate bonds provided by three rating agencies			0.472*** (0.077)

Table 12 reports that the numbers in the parentheses are standard errors.

*, **, *** denote that the coefficient is statistically significant at the 10%, 5%, 1% levels respectively.

Overall, the empirical results show that the dual-rating regulation is best for preventing rating inflation and improving rating quality.

## Conclusions and research prospect

In this study, we investigated the regulatory effects of different rating regulations on corporate bond ratings. Using the dual reputational rating shopping and the panel ordered logit models, we draw the following conclusions:

First, when investors’ and regulators’ penalty rates are low, the single-rating regulation is effective in improving rating quality. However, because of low reputation effect, the dual-rating regulation is ineffective in controlling inflation.

Second, when investors’ and regulators’ penalty rates are high, the regulatory effect of the dual-rating regulation is better than that of the single-rating regulation.

Third, the comparison of the effects of different rating regulations on rating upgrades shows that rating agencies are more stringent in providing rating upgrades under the single-rating and dual-rating regulations compared to the multi-rating regulation.

Fourth, the comparison of the effects of different rating regulations on rating downgrades suggest that the three rating regulations are effective in decreasing rating deflation. The regulatory effect of the dual-rating regulation is the best, and rating agencies can improve rating quality under this regulation.

Fifth, the effort of rating agencies is the highest under the dual-rating regulation, and rating agencies can improve their efforts to provide more rating information under different rating regulations.

Finally, the probability of rating defaults is the lowest under the dual-rating regulation, and rating agencies can decrease rating defaults under different rating regulations.

Based on the current state of the Chinese rating industry, we can develop many new theoretical models to analyze the available conditions of different rating regulations. Meanwhile, given the development of data mining, we will utilize the latest empirical models to discuss the regulatory effect of rating regulations on rating quality.

## Supporting information

S1 Data This is the data set for the research. (XLSX)

S2 Data This is the data set for the research. (XLSX)

S3 Data This is the data set for the research. (XLSX)

S1 Appendix This is the supplement of the research. (DOCX)
