# Supplementary material for: Can the dual-rating regulation improve the rating quality of Chinese corporate bonds?
Source: PLoS One. 2021 Dec 2;16(12):e0259759. doi: 10.1371/journal.pone.0259759 (PMC8638972; doi:10.1371/journal.pone.0259759)
Supplement: S1 Appendix — (DOCX) [file pone.0259759.s004.docx]

**Proof 1 of proposition 1:**

Letting$R_{1}>R_{2}>R_{3}$, we have:

In this assumption, the investors’ penalty rate$\rho_{RA}\leq1$. Therefore, we get$\rho_{RA}\in(1-m,1]$.

$$c_{1}=(1-\rho_{RE})[c_{A}+\sum_{t=2}^{\infty} \rho_{RE}^{t-1}c_{A}+\sum_{t=1}^{\infty} \rho_{RE}^{t-1}c_{M}]=c_{A}+c_{M}$$

$$c_{2}=(1-\rho_{RE})[2c_{A}+\sum_{t=1}^{\infty} \rho_{RE}^{t-1}2c_{M}]=2c_{A}\cdot(1-\rho_{RE})+2c_{M}$$

Letting$c_{2}>c_{1}$and$c_{A}>c_{M}$, we have$\rho_{RE}\in(0,\frac{c_{A}+c_{M}}{2c_{A}})$.

**Proof 2 of proposition 2:**

Letting$R_{4}>R_{5}$, we have:

In this assumption, the investors’ penalty rate$\rho_{RA}\leq1$. Therefore, we get$\rho_{RA}\in(\frac{2(\alpha_{i}-m)}{2\alpha_{i}-m},1]$

Letting$c_{1}>c_{2}$and$c_{A}>c_{M}$, we have$\rho_{RE}\in(\frac{c_{A}+c_{M}}{2c_{A}},1)$.
